# Beneath the Surface: A Case Report Revealing a Rare Cystic Neoplasm Hidden Within a Pseudocyst in the Pancreas and a Scientific Literature Review

**DOI:** 10.7759/cureus.64158

**Published:** 2024-07-09

**Authors:** Neelesh Shrivastava, Richa Shukla, Kumari Nutan

**Affiliations:** 1 Department of Surgical Oncology, All India Institute of Medical Sciences, Bhopal, Bhopal, IND

**Keywords:** pancreatic pseudocyst (ppc), surgical case reports, superior mesenteric artery (sma), total pancreaticoduodenectomy, solid pseudopapillary neoplasm

## Abstract

Solid pseudopapillary neoplasms (SPNs) of the pancreas are rare tumours with distinctive clinicopathological features. We present a case of a 51-year-old female with a large cystic neoplasm involving the entire pancreas, initially presenting with abdominal pain. Diagnostic imaging revealed a well-defined heterogeneously enhancing mixed solid cystic lesion in the pancreas. Surgical exploration confirmed a lesion in the entire pancreas, prompting total pancreatectomy with duodenectomy. Postoperative histopathology and immunohistochemistry supported the diagnosis of SPN. Herein, we discuss SPN's clinical presentation, diagnostic challenges, surgical management, and pathological characteristics.

## Introduction

Solid pseudopapillary neoplasms (SPNs) of the pancreas are rare tumours, accounting for only 1-2% of pancreatic neoplasms, with a notable predominance in females. These tumours often remain asymptomatic and can grow to a considerable size by the time of diagnosis [1]. Despite their size, SPNs generally have a favourable prognosis following surgical resection [2]. The definitive diagnosis of SPNs typically relies on pathological and cytological evaluation [3]. However, despite advancements in imaging techniques, preoperative diagnosis remains challenging, and the underlying pathophysiology of SPNs continues to be a subject of debate. We documented a rare case of SPN of the pancreas, characterised by a clinical presentation mimicking pancreatic pseudocyst, posing challenges in preoperative diagnosis.

## Case presentation

**Figure 1:**
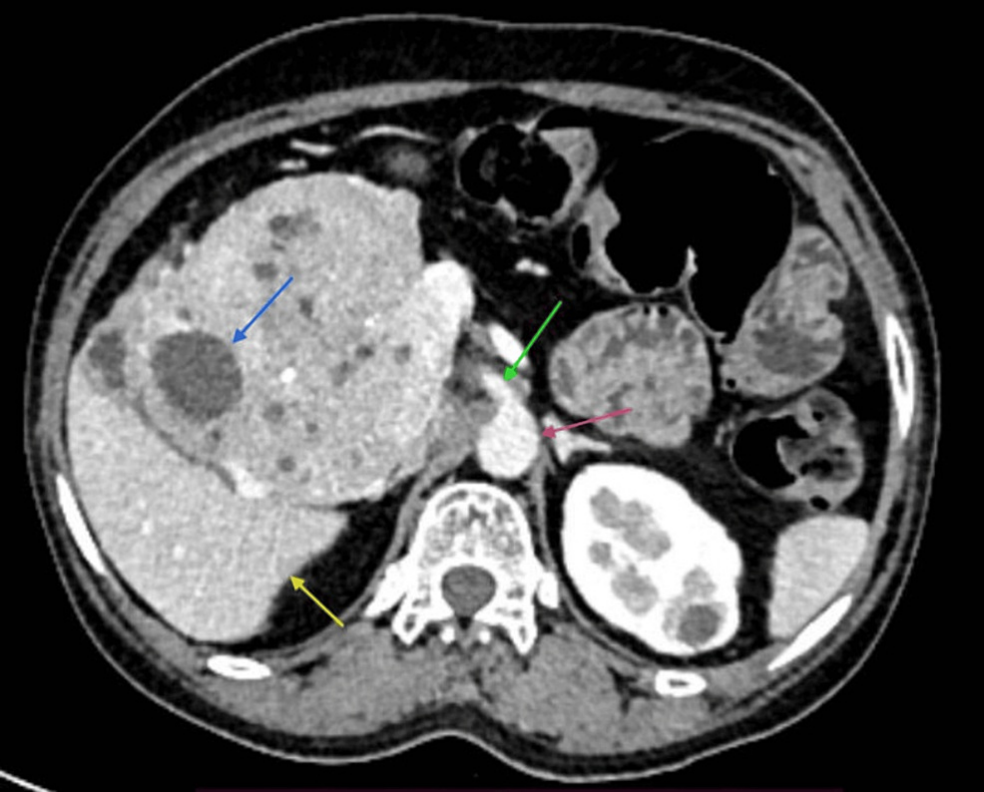
Triple-phase abdomen CT showing cystic pancreatic lesion. The red arrow points out the aorta, the green arrow points to the superior mesenteric artery, the blue arrow points to a cystic lesion of the pancreas, and the yellow arrow points to the liver.

A 51-year-old female presented with a one-year history of chronic epigastric pain. Physical examination revealed a palpable mass in the epigastrium extending to the right hypochondrium. Imaging studies, including triple-phase computed tomography (CT) of the abdomen and pelvis, demonstrated a large cystic lesion originating from the pancreas head with no differentiation from other parts of the pancreas and without the involvement of portal vein, superior mesenteric vein (SMV), superior mesenteric artery (SMA), and common hepatic artery (CHA) (Figure 1).

**Figure 2:**
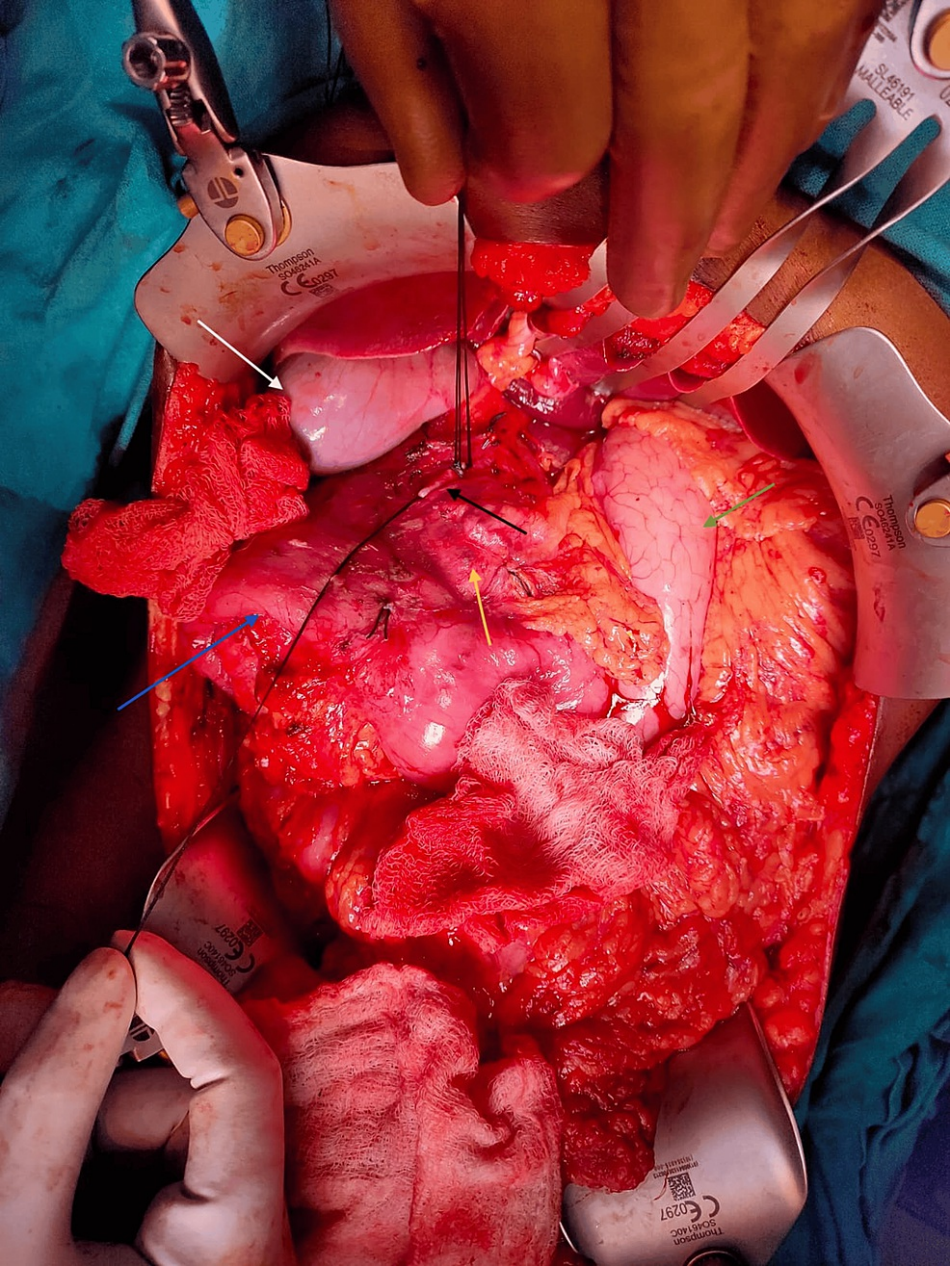
Intraoperative picture showing the cystic neoplasm. The white arrow points out the gallbladder, the black arrow shows the gastroduodenal artery (ligated), the yellow arrow points to a cystic pancreatic lesion, the blue arrow shows the duodenum, and the green arrow shows the stomach.

According to the patient's history, she had episodes of recurrent abdominal pain, which were supposed to resolve on their own without any specific medication. Her tumour markers were in the normal range, including carbohydrate antigen 19-9 (CA 19-9) at 20 U/ml and carcinoma embryonic antigen (CEA) at 2 ng/ml, as well as normal serum amylase and lipase. Given the cystic nature of the lesion and routine tumour marker, diagnosis of pancreatic pseudocyst was most likely. Although the patient was from a poor socioeconomic background and due to the unavailability of endoscopic ultrasound in our institution, further evaluation of the lesion was not possible. We then decided to proceed with exploratory laparotomy. The patient was taken up for surgery. Surgical exploration identified a lesion involving the pancreatic head, body, and tail, necessitating total pancreatectomy with duodenectomy (Figure 2). Surgery was completed in 10 hours with minimal blood loss (<200 ml).

**Figure 3:**
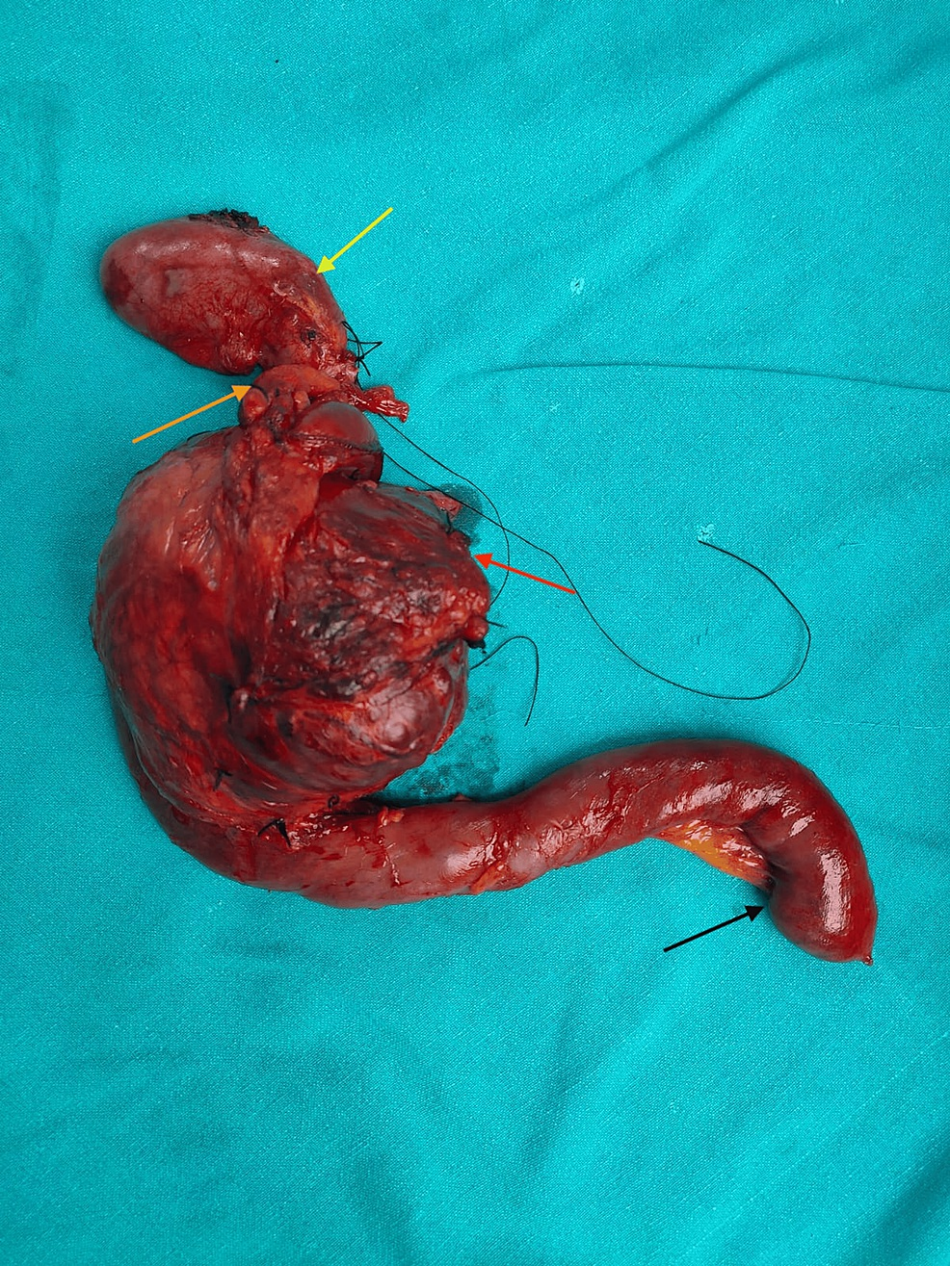
Postoperative specimen of the resected tumour. The yellow arrow points out the gallbladder, the bright orange arrow shows the common bile duct, the dark orange arrow shows a cystic neoplasm of the pancreas with no anatomical differentiation, and the black arrow points to the second part of the duodenum.

Figure 3 represents the postoperative specimen, which shows no differentiation between the different anatomical landmarks of the pancreas. Postoperatively, the patient recovered well and was discharged on the 10th day without any surgical complications. Histopathological examination of the excised specimen revealed characteristic morphological features consistent with SPN, further supported by positive immunohistochemical staining. She was discussed in the tumour clinic and, given the specific nature of the disease, it was planned to observe her every three months. The patient was last followed up one month before and is doing well.

## Discussion

Review of the literature

SPNs of the pancreas, also known as Frantz tumours, are rare tumours that predominantly affect young females. In our case, the patient belonged to the middle age group as her age was 51 years; however, compared to existing the literature, which shows female predominance, our patient was also female. Indolent behaviour, distinct histological features, and a favourable prognosis following surgical resection characterise these neoplasms. In our case, the patient has been suffering from recurrent attacks of upper abdominal pain for the last year and has a history of spontaneous resolution of symptoms without any major treatment, which supports the indolent nature of the disease. However, despite their relative rarity, SPNs pose diagnostic and management challenges due to their varied clinical presentations and imaging characteristics [1]. In our case, the patient was presented with a mass in the epigastric region with no symptoms of obstructive jaundice, and at first instance, it was supposed to be a gastric mass. Even on abdomen contrast-enhanced computed tomography, the diagnosis of pseudocyst of the pancreas was suspected. This supported the diagnostic challenge for such types of rare neoplasms.

This case is unique because mimicking an SPN as a pseudocyst is rare and hardly reported in the literature.

The cellular origin and pathogenesis of SPNs remain areas of ongoing investigation. Historically, SPNs were believed to arise from pluripotent cells of pancreatic ducts or acini, given their diverse histological patterns. However, recent molecular studies suggest a possible origin from primitive cells with both exocrine and endocrine differentiation potential [2]. Mutations in the CTNNB1 gene, encoding β-catenin, are commonly observed in SPNs and are thought to play a pivotal role in tumorigenesis by dysregulating the Wnt/β-catenin signalling pathway [3].

Table 1 shows the compiled studies done for these tumours. Such tumours are rare, and limited literature is available on such lesions regarding workup and management.

**Table 1:** Compiled studies on solid pseudopapillary neoplasms.

Article	Authors	Year	Journal	Results
Solid-pseudopapillary tumor of the pancreas: a typically cystic carcinoma of low malignant potential	Klimstra et al. [1]	2000	Seminars in Diagnostic Pathology	Describes solid-pseudopapillary tumour as a typically cystic carcinoma of low malignant potential.
Solid pseudopapillary tumor of the pancreas: A review of 553 cases in Chinese literature	Yu et al. [2]	2010	World Journal of Gastroenterology	Surgery, including local resection and pancreatectomy, is the mainstay treatment, resulting in a high five-year survival rate (96.9%) despite occasional recurrence and metastasis, indicating that solid pseudopapillary tumour of the pancreas is typically indolent, representing a low-grade curable malignancy.
Solid Pseudopapillary Tumor of the Pancreas: A Case Report	Chowdhury et al. [3]	2019	Mymensingh Medical Journal: MMJ	A solid pseudopapillary tumour (SPT) exhibits low-grade malignant potential but has excellent post-surgical curative rates and rare metastasis. Pathological and cytological evaluation remains the gold standard for definitive diagnosis. Additionally, it predominantly affects young females.
Pancreatic neuroendocrine tumor and solid-pseudopapillary neoplasm: Key immunohistochemical profiles for differential diagnosis	Ohara et al. [4]	2016	World Journal of Gastroenterology	Distinguishing between pancreatic neuroendocrine tumour (NET) and solid pseudopapillary neoplasm (SPN), E-cadherin, chromogranin A, and β-catenin are highlighted as the most valuable markers for immunohistochemical differentiation.
Diagnosis and treatment of solid pseudopapillary tumor of the pancreas: experience of one single institution from Turkey	Yagcı et al. [5]	2013	World Journal of Surgical Oncology	Solid pseudopapillary neoplasm (SPN) primarily affects young women with a favourable prognosis even in the presence of distant metastasis. While surgical resection is typically curative, close follow-up is recommended to detect local recurrence or distant metastasis and select appropriate therapeutic interventions.
Solid pseudopapillary neoplasm of the pancreas: clinicopathologic feature, risk factors of malignancy, and survival analysis of 53 cases from a single center	Song et al. [6]	2017	BioMed Research International	The presence of an incomplete capsule in solid pseudopapillary neoplasm (SPN) may indicate malignancy and serve as a prognostic indicator for disease-specific survival, prompting surgeons to consider a more radical resection approach in such cases.

Clinically, SPNs often present as significant, well-circumscribed masses with variable cystic and solid components on imaging studies. While characteristic radiological findings such as peripheral calcifications, haemorrhage, and capsule formation can aid the diagnosis, in our case, on radiological imaging, no calcification, haemorrhage, or capsule formation was reported, which is against the common radiological features of SPN. SPNs can mimic other pancreatic neoplasms, including mucinous cystic neoplasms and pancreatic neuroendocrine tumours [4]. In our case, it was assumed a pseudocyst, which is also against the common radiological diagnosis of SPNs. Consequently, definitive diagnosis relies on histopathological examination, with characteristic features, including pseudopapillary structures, hyalinised stroma, and nuclear β-catenin expression on immunohistochemistry [2].

Surgical resection with negative margins remains the cornerstone of treatment for SPNs. Given their low-grade malignant potential, complete excision usually results in an excellent prognosis, with reported long-term survival rates exceeding 95%. In our case, we proceeded with pancreaticoduodenectomy, which included total pancreatectomy given the entire pancreas was involved and no anatomical part differentiation of the pancreas was identifiable. However, the optimal surgical approach (e.g., enucleation vs. formal resection) and the role of lymphadenectomy in SPN management remain topics of debate, particularly in the context of minimizing operative morbidity while ensuring oncological efficacy [5]. In our case, we proceeded with wide resection with a negative margin with no lymph node dissection.

The radiological diagnosis is often challenging for SPN. Sometimes it can mimic pseudocysts, as in our case, and this is a unique presentation, which can give a message that in a cystic lesion or pseudocystic lesion with prolonged history, it is wishful to rule out malignancy by serial evaluation of lesion with endoscopic ultrasound and biopsy as well as with tumour marker. In the radiological image, SPN typically represents a wide-ranging appearance from solid to cystic, a well-capsulated mass, with solid and cystic components [6].

Despite the generally favourable outcomes associated with SPNs, some patients may experience disease recurrence or metastasis following resection. Factors predictive of recurrence include tumour size > 5 cm, vascular invasion, and incomplete surgical resection. Therefore, long-term surveillance with regular imaging studies is recommended to detect recurrence early and facilitate prompt intervention [7].

## Conclusions

SPNs of the pancreas present a diagnostic challenge due to their rarity and unique characteristics; as in our case, by detailed investigation, the lesion appeared as a pseudocyst at initial presentation. Endoscopic ultrasound has emerged as the preferred method for investigating such lesions, allowing for guided biopsies and facilitating preoperative histopathological diagnosis. Unlike other pancreatic tumours, SPNs do not typically respond to chemotherapy or radiation therapy. Surgical resection with negative margins remains the cornerstone of treatment, offering excellent patient outcomes. With this case report, we can understand that if a cystic lesion of the pancreas, especially pseudocyst, is presented in clinical practice with long-duration symptoms, SPNs or another cystic neoplasm of the pancreas should be ruled out.
